# Investigating Radiotherapy Response in a Novel Syngeneic Model of Prostate Cancer

**DOI:** 10.3390/cancers12102804

**Published:** 2020-09-29

**Authors:** Charles M. Haughey, Debayan Mukherjee, Rebecca E. Steele, Amy Popple, Lara Dura-Perez, Adam Pickard, Mehjabin Patel, Suneil Jain, Paul B. Mullan, Rich Williams, Pedro Oliveira, Niamh E. Buckley, Jamie Honeychurch, Simon S. McDade, Timothy Illidge, Ian G. Mills, Sharon L. Eddie

**Affiliations:** 1 Patrick G Johnston Centre for Cancer Research, Queen’s University, Belfast BT9 7AE, UK; charles.haughey@nds.ox.ac.uk (C.M.H.); rebecca.steele@icr.ac.uk (R.E.S.); laraduraperez@gmail.com (L.D.-P.); adam.pickard@manchester.ac.uk (A.P.); s.jain@qub.ac.uk (S.J.); P.Mullan@qub.ac.uk (P.B.M.); rich.williams@qub.ac.uk (R.W.); n.obrien@qub.ac.uk (N.E.B.); S.McDade@qub.ac.uk (S.S.M.); 2 Nuffield Department of Surgical Sciences, John Radcliffe Hospital, University of Oxford, Oxford OX3 9DU, UK; 3 Targeted Therapy Group, Division of Cancer Sciences, Faculty of Biology Medicine and Health, The University of Manchester, Manchester M13 9PL, UK; debayan.mukherjee@manchester.ac.uk (D.M.); apopple@microbiotica.com (A.P.); mehjabinpatel94@hotmail.com (M.P.); Jamie.Honeychurch@manchester.ac.uk (J.H.); 4 The Breast Cancer Now Toby Robins Breast Cancer Research Centre, The Institute of Cancer Research, London SM2 5NG, UK; 5 Wellcome Centre for Cell Matrix Research, University of Manchester, Manchester M13 9PL, UK; 6 The Christie Hospital Foundation Trust, Manchester M20 4BX, UK; Pedro.Oliveira@christie.nhs.uk; 7 School of Pharmacy, Queen’s University Belfast, Belfast BT9 7BL, UK

**Keywords:** tumour microenvironment, syngeneic model, prostate cancer, radiotherapy, preclinical modelling, myeloid-derived suppressor cells

## Abstract

**Simple Summary:**

Pre-clinical models are required to develop new therapeutics to improve patient care. In the prostate cancer field, significant progress has been made in the development of in vivo models but with a predominant focus on transgenics, which are time and cost prohibitive. Conversely, other available models do not closely resemble patient disease and tumour immune microenvironment. In this study, a new graft-based model is described, using a cell-line derived from a transgenic: the DVL3 model. Grafts using the DVL3 cells retain the pathological and immunological features of localized clinical disease, whilst genetically the model is sustained by poor prognosis drivers of disease progression. Irradiating tumours post-engraftment leads to remodeling of the tumour immune microenvironment and increased expression of genes associated with nucleic acid sensing pathways and the type I interferon response. This paper establishes this model as resource for the pre-clinical characterization of new prostate cancer therapies and biological responses to treatment.

**Abstract:**

The prostate cancer (PCa) field lacks clinically relevant, syngeneic mouse models which retain the tumour microenvironment observed in PCa patients. This study establishes a cell line from prostate tumour tissue derived from the *Pten^−/−^/trp53^−/−^* mouse, termed DVL3 which when subcutaneously implanted in immunocompetent C57BL/6 mice, forms tumours with distinct glandular morphology, strong cytokeratin 8 and androgen receptor expression, recapitulating high-risk localised human PCa. Compared to the commonly used TRAMP C1 model, generated with SV40 large T-antigen, DVL3 tumours are immunologically cold, with a lower proportion of CD8+ T-cells, and high proportion of immunosuppressive myeloid derived suppressor cells (MDSCs), thus resembling high-risk PCa. Furthermore, DVL3 tumours are responsive to fractionated RT, a standard treatment for localised and metastatic PCa, compared to the TRAMP C1 model. RNA-sequencing of irradiated DVL3 tumours identified upregulation of type-1 interferon and STING pathways, as well as transcripts associated with MDSCs. Upregulation of STING expression in tumour epithelium and the recruitment of MDSCs following irradiation was confirmed by immunohistochemistry. The DVL3 syngeneic model represents substantial progress in preclinical PCa modelling, displaying pathological, micro-environmental and treatment responses observed in molecular high-risk disease. Our study supports using this model for development and validation of treatments targeting PCa, especially novel immune therapeutic agents.

## 1. Introduction

Prostate cancer (PCa) is the most common cancer in men and the fifth leading cause of cancer deaths [1]. It is estimated that 1.3 million new cases of PCa were diagnosed worldwide in 2018 alone [1]. The vast majority of PCa (91%) is localised disease at diagnosis [2], and can be treated with a range of therapeutic modalities including surgery, androgen-deprivation therapy (ADT) and radiotherapy (RT). Unfortunately, roughly 30% of high-risk localised PCa will develop into aggressive metastatic disease [3], with limited treatment options. The current standard of care for these patients is ADT, but this has many adverse side effects and has a median relapse rate of only 11 months [4].

As a result, efforts have focused on the use of RT, as it can be utilised to treat both localised and metastatic disease, with comparable patient outcomes to radical prostatectomy [5]. However, one major challenge in enhancing RT response, by combining this modality with other treatments, is the paucity of appropriate preclinical PCa models in which to test these combinations. Immune comprised mice are required for xenograft models, thus failing to recapitulate the patient tumour microenvironment (TME) and the critical role the immune component plays in both therapeutic response and relapse of disease.

The most commonly used immune-competent model is the TRAMP C1 murine cell line, which can be syngenically, subcutaneously grafted to generate more uniform, accessible prostate tumours [6]. The TRAMP C1 model was generated by engineered expression of viral SV40 large T-antigen. Unfortunately, TRAMP C1 allografts develop neuroendocrine tumours which are rare clinically, rather than adenocarcinomas which are most often seen in PCa patients [7]. 

Consequently, novel PCa models that more accurately recapitulate human tumours and the surrounding TME are urgently required, in order to continue to develop and improve PCa treatment. *PTEN* deletion occurs in ~20% of localized PCa, and is implicated in RT failure [8,9], however, an engraftable mouse syngeneic model with *Pten* deletion, which can be utilised to investigate host response to radiotherapy has long been lacking. In this study we have developed a syngenic model from the *Pten^−/−^/trp53^−/−^* transgenic mouse tumour [10]; the DVL3 cell line (derived from tumour formed from the dorsal, ventral and lateral prostate lobes. These lobes are most similar to the peripheral zone of the human prostate where 75–85% of adenocarcinomas originate [11]; whereas, the anterior lobe of the mouse prostate is considered analogous to the central zone which rarely develops cancer in the human prostate [12].

DVL3 cells develop tumours in immune competent, C57BL/6 mice that retain morphological, lineage and immune characteristics of localised, high-risk PCa. These tumours respond to RT, retain androgen receptor (AR) expression and sensitivity to androgens, and display an immune ‘cold’ phenotype with tumours being poorly infiltrated by T-cells, and heavily infiltrated with myeloid cells, which is primarily driven by *Pten* loss [13]. Clinically, human prostate cancers are broadly classified as non-T-cell inflamed/ ‘cold’ tumours [14], and PTEN deficiency is associated with an immunosuppressive TME [15]. The DVL3 model accurately mimics both patient disease and TME and is therefore ideal for future pre-clinical evaluation of novel treatment combinations including immune therapeutic agents.

## 2. Results

### 2.1. DVL3 Cell Engraftment in Immunocompetent Mice Results in Tumour Formation, which Accurately Models Human Prostate Adenocarcinoma 

**Figure 1:**
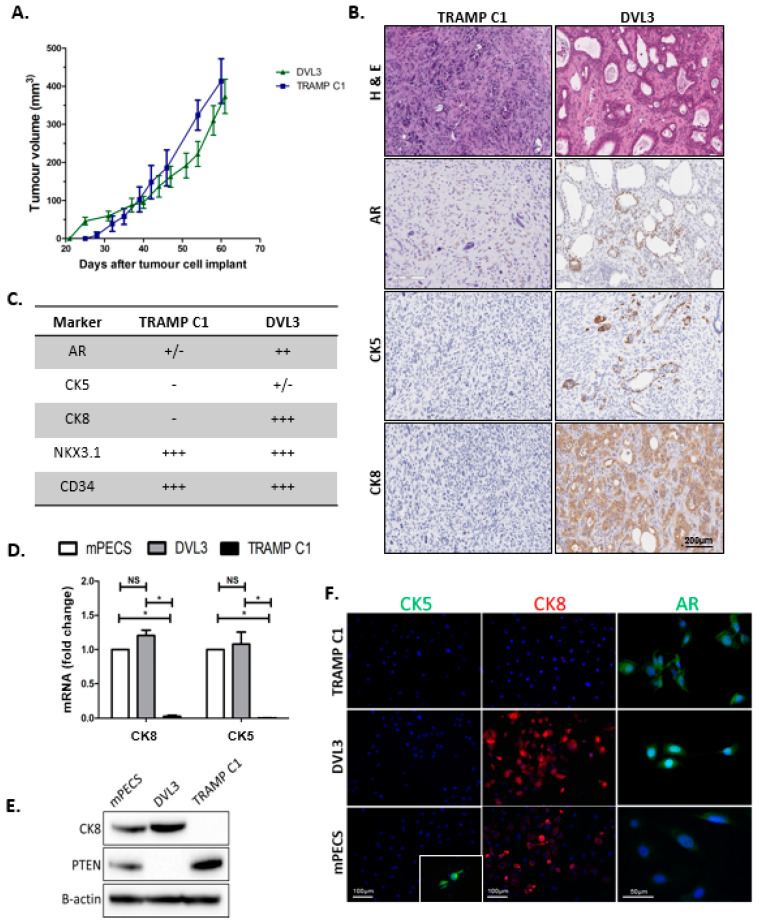
DVL3 syngeneic tumours replicate patient disease. (**A**) DVL3 tumour growth (Green) is comparable to TRAMP C1 (Blue), *n* = 5–8 mice per group. Both models take ~4 weeks to generate substantial tumours. The mPEC model of normal prostate epithelium did not generate tumours (data not shown) (**B**) DVL3 tumours develop heterogeneous glandular morphology graded at Gleason 7, whereas TRAMP C1 tumours were undifferentiated with neuroendocrine features (H&E). DVL3 also expressed clinical prostate cancer markers; androgen receptor (AR), cytokeratin 5 (CK5) and cytokeratin 8 (CK8). TRAMP C1 expressed no CK8 or CK5 and less AR than DVL3 tumours, with complete absence of AR noted in some tumours. (**C**) Summary of histological evaluation of prostate specific markers in DVL3 and TRAMP C1 prostate tumours (− indicates absence, +/– presence in only some of tumour samples, and + indicates the level of positive expression). Both DVL3 and TRAMP C1 express NKX3.1 and CD34 (Supplementary Figure S2). (**D**) Both novel cell models (mPEC and DVL3) express CK5 and CK8 mRNA at significantly higher levels than TRAMP C1 cells, as analysed via qRT-PCR (*n* = 3 biological replicates per cell type). mRNA expression was normalised to β-actin expression and shown as a fold change relative to mPECs transcript levels. (**E**) Expression of CK8 (luminal marker, 60 kDa) was also evident at the protein level. Importantly, DVL3 cells demonstrate loss of the clinically relevant tumour suppressor, Pten (55 kDA). B-actin provided as a loading control (47 kDa). (**F**) Immunocytochemical staining was consistent with qRT-PCR and western blotting results for CK8 (Red) and AR (Green). CK5 protein (Green) was not detectable in any of the models (MCF7 cells provided as a positive control, inset). Dapi (Blue) used as a counterstain. Data represents mean ± SEM. * denotes *p* ≤ 0.05, NS denotes non-significant, as determined by Student’s *t*-test. Immunohistochemistry and western blotting are representative of *n* = 3 biological replicates.

Murine cell lines were generated via spontaneous immortalisation of normal prostate epithelium (mPECs) and prostate tumours (DVL3) (Supplementary Figure S1). To establish tumorigenic potential, both mPEC and the DVL3 cells were subcutaneously implanted into wild-type C57BL/6 male mice, as all cell lines were originally generated from the C57BL/6 strain. Engrafted tumour growth rate was compared to the established TRAMP C1 model. Mice engrafted with mPEC cells did not develop any sign of disease after 12 weeks (data not shown), consistent with their status as untransformed, but spontaneously immortalised wild-type prostate epithelial cells. DVL3 tumours grew at a similar rate to the TRAMP C1 model, with measurable tumour established after 4 weeks post-inoculation (Figure 1A). DVL3 tumours displayed heterogeneous pathology with neoplastic, glandular structures akin to human acinar adenocarcinoma (Figure 1B, Supplementary Figure S2A). Some regions of adenosarcoma were observed in larger, terminal endpoint tumours as previously reported arising from *Pten^−/−^/trp53^−/−^* Pb-Cre4 mice [10]. In contrast, TRAMP C1 tumours were uniformly undifferentiated and lacked glandular morphology (Figure 1B). 

PCa arises from glandular epithelium of the prostate, and retains expression of classical prostate markers including cytokeratins 5 and 8 (CK5 and CK8, basal and luminal epithelial markers respectively) [16]. Immunohistochemical staining revealed that the DVL3 tumours highly expressed CK8 throughout the entire tumour, particularly in the cells surrounding the lumen of glandular structures (representative images in Figure 1B,C with further images in Supplementary Figure S3). DVL3 tumours also had regions of CK5 positivity, with varied expression between the tumours. In comparison the TRAMP C1 tumours lacked the expression of both CK8 and CK5 (Figure 1B,C).

Androgen receptor (AR) was present in the nucleus of cells throughout DVL3 tumours but was most abundant in clustered regions lining the lumen of glandular structures. Conversely, TRAMP C1 tumours had notably less AR staining likely due to fewer glandular structures, with some tumours lacking AR expression entirely (Figure 1B,C, Supplementary Figures S2 and S3). To investigate if AR within the tumours was active, a downstream target of the AR, NKX3.1 expression was evaluated. NKX3.1 is prostate-specific protein, and thus used to identify PCa metastatic disease and is also known to be lost in castrate-resistant PCa [17,18]. Both the DVL3 and the TRAMP C1 tumours were positive for NKX3.1 throughout the tumour section (Supplementary Figures S2B and S3). Expression of CD34, which highlights endothelial cells, was also investigated, with no notable differences observed in staining intensity between the models (Supplementary Figure S2B).

Characterisation of mPEC and DVL3 cell lines was also performed to establish expression in serially passaged cell lines in vitro. qRT-PCR revealed significantly higher mRNA expression of CK5 and CK8 in mPEC and DVL3 cells compared to the TRAMP C1 cells (Figure 1D). Protein expression of CK8 was also notably higher in mPEC and DVL3 cells compared to TRAMP C1 and the DVL3 cells did not express Pten, in keeping with their origin (Figure 1E, densitometry provided in Supplementary Figure S4). All models expressed AR at both the mRNA and protein level (Supplementary Figures S2C,D and S4). Although expression of AR was higher in TRAMP C1 cells, DVL3 responded similarly to the AR antagonist (enzalutamide), as demonstrated in vitro using cell viability assay (Supplementary Figure S2E). The expression of CK8 and AR was also validated using immunofluorescence staining (Figure 1F). Interestingly, CK5 protein expression was not detectable in any of the prostate cell models when examined via immunocytochemistry (Figure 1F). 

### 2.2. DVL3 Tumours Have Immunosuppressive Microenvironment Similar to Human Prostate Adenocarcinomas

**Figure 2:**
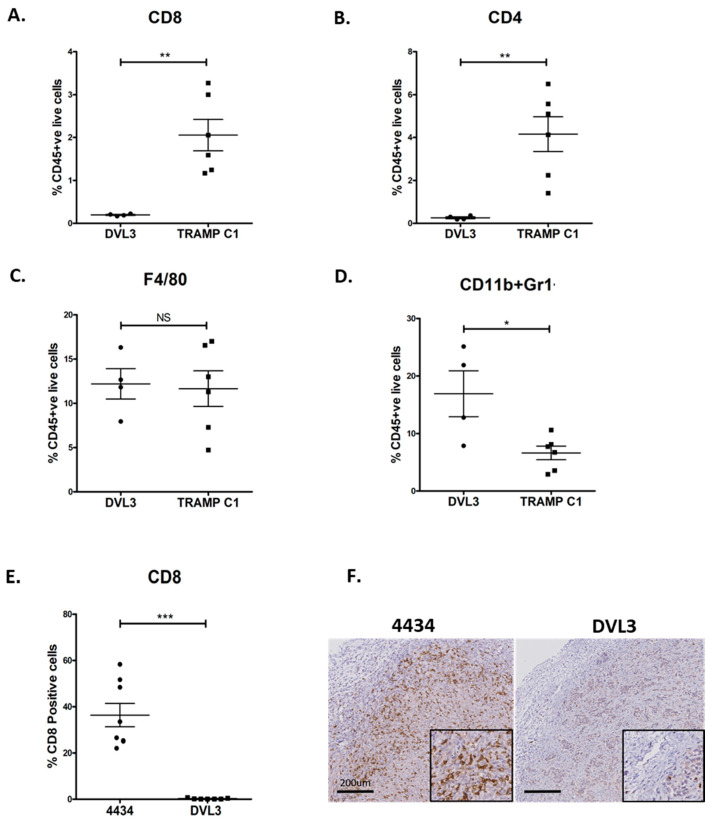
The DVL3 model generates immunosuppressive, ‘cold’ tumours similar to the TME seen clinically in prostate cancer. Flow cytometry was performed on DVL3 and TRAMP-C1 subcutaneous, syngeneic tumours taken 4-6 weeks post engraftment or when they reached the treatment size (100–200 mm^3^). DVL3 tumours had significantly fewer (**A**) cytotoxic (CD8+) and (**B**) helper T cells (CD4+) when compared with the TRAMP-C1 model. (**C**) There was no difference in the proportion of macrophages (F4/80) between the two models. (**D**) However, the population of myeloid derived suppressor cells (MDSCs, CD11b+Gr1+) was significantly increased in the DVL3 compared to TRAMP-C1 tumours. (**E**) Quantification of immunohistochemical staining for CD8+ T-cells in inflamed syngeneic murine (4434 Braf^4006E^) melanoma model compared to the non-immunogenic DVL3 murine prostate cancer model shown as a percentage of total cells as quantified by haematoxylin. (**F**) Representative IHC staining comparing 4434 (Braf^4006E^) melanoma vs DVL3 prostate tumour demonstrating lack of CD8+T-cells within the tumour microenvironment in the DVL3 tumours. In 4434 (Braf^4006E^) melanoma model CD8+ T-cells are uniformly distributed, including the central of the tumour. Data represents mean ± SEM of *n* = 4–9 independent murine tumors as indicated by points on each graph. * denotes *p* ≤ 0.05, ** denotes *p* ≤ 0.01, *** denotes *p* < 0.001 as determined by Mann-Whitney, non-parametric testing.

Human prostate cancers are broadly classified as non-T-cell inflamed/‘cold’ tumours with dysfunctional or suppressed tumour infiltrating lymphocytes (TILs) [14]. Pten deficient prostate cancers are also associated with an immunosuppressive TME [15]. Having established that DVL3 tumours (*Pten^−/−^/trp53^−/−^*) retained expression of several key markers of human disease, the immunological characteristic of the TME were assessed compared to the TRAMP-C1. Flow cytometry was performed for T cells (CD4+, CD8+), macrophages (F4/80), and myeloid-derived suppressor cells (MDSC), identified as dual (Gr1+/CD11b+) cells in dissociated DVL3 and TRAMP C1 (gating strategy demonstrated in Supplementary Figure S5)**,** at similar size/ tumor volume for baseline immune profiling (Supplementary Figure S6A). The DVL3 tumours have a lower proportion of both cytotoxic CD8 + T-cells (0.187% vs. 2.05%, *p* = 0.01) and CD4 + T-cells (0.36% vs. 4.31%, *p* = 0.01) compared to the TRAMP C1 tumours (Figure 2A,B). In contrast, both tumour lines have comparable level of macrophage (F4/80+) cells (Figure 2C). The DVL3 tumours have a higher proportion of MDSCs (CD11b+/ Gr1+), compared to the TRAMP C1 tumour (16.90% vs 6.61%, *p* = 0.04, Figure 2D).

Interestingly, the DVL3 tumours have higher proportion of MDSCs (CD11b+/Gr1+ cells) compared to other syngeneic mouse models, such as 4T1 (breast cancer) CT26 (colorectal cancer) and 4434 (BRAF^V600E^ melanoma) models (*p* = 0.003. 0.01, and 0.01 respectively, Supplementary Figure S6B,C). Comparison to other syngeneic models was performed using tumours that had reached terminal endpoint and thus were larger in volume. Notably, these data suggest that in the DVL3 model, irrespective of the tumour size, the relative ratio of (CD11b +/Gr1 +) cells within the leukocyte compartment is significantly higher and increases over time or as the tumour develops.

We also evaluated the CD8+ T cell using immunohistochemistry in DVL3 tumours compared to the 4434 melanoma model, which are immunologically active, ‘hot’ tumours [19] (Figure 2E). In agreement with the flow cytometry data (Figure 2A), immunohistochemistry demonstrates the DVL3 tumours are immunologically ‘cold’ having very few basal CD8+ T cells, that were sparsely distributed primarily around the tumour edge, but absent in the centre or around the tumour epithelium/ glandular structures. 

In comparison the syngeneic murine melanoma 4434 (BRAF^V600E^) model had a greater proportion of CD8 + T-cells (*p* < 0.001), which were uniformly distributed, including in the central region of the tumour (Figure 2E,F). These data demonstrate that murine DVL3 prostate tumours have immunologically inactive/ non-inflamed phenotype, with the TME primarily compromising of myeloid cells, but, lacking TILs population similar to the advanced human disease.

### 2.3. Fractionated Radiotherapy Leads to Marginal Growth Delay and Alters the Local Tumour Immune Microenvironment 

**Figure 3:**
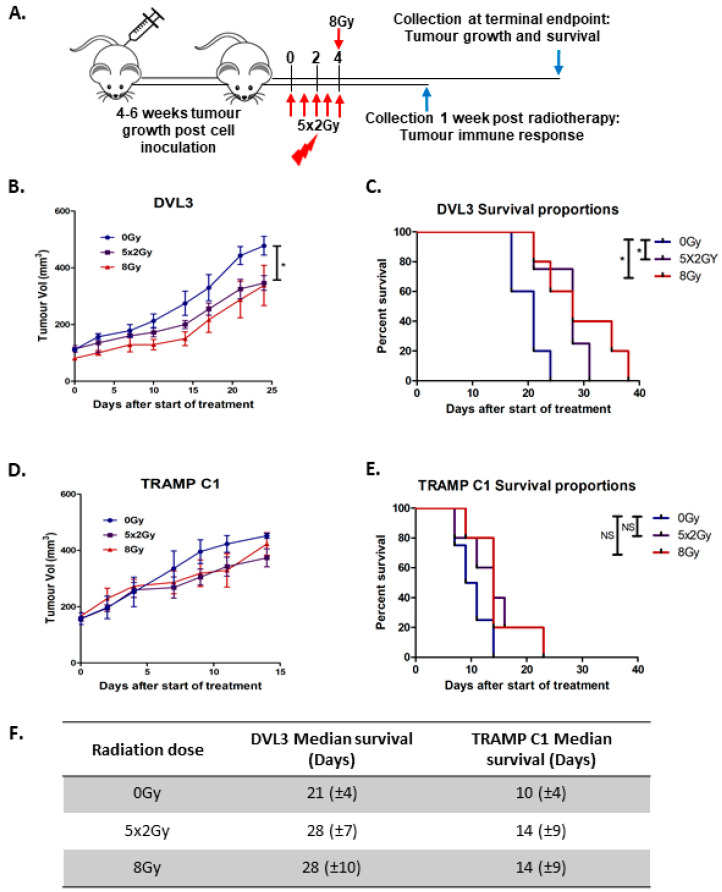
DVL3 tumours respond and are more sensitive to radiotherapy than the resistant TRAMP C1 model. (**A**) DVL3 or TRAMP C1 cells were engrafted into immune competent mice and allowed to reach a tumour volume of 100–200 mm^3^. Mice were randomly assigned to no treatment (0 Gy) (Blue), fractionated dose (5 × 2 Gy) (Purple) or single high dose (8 Gy) (Red) radiotherapy (RT) group. A cohort of mice were sacrificed one week after radiotherapy to examine immune response and a further cohort was left to terminal endpoint to examine tumour growth (Figure 1A) and survival. (**B**) DVL3 tumours were sensitive to fractionated RT but no difference was seen with single high dose RT. (**C**) Kaplan Meier curves showing survival in mice engrafted with DVL3 tumour cells was improved for both treatment regimens (5 × 2 Gy and 8 Gy) compared to for mice treated with 0 Gy (5 × 2 Gy *p* = 0.0255 and 8 Gy *p* = 0.0386). (**D**) TRAMP C1 tumours did not respond to radiotherapy. (**E**) There was no change in survival with radiation in the mice engrafted with TRAMP C1 cells treated with RT. (**F**) Summary of timeline for DVL3 and TRAMP C1 tumours treated with RT, demonstrating DVL3 tumours are more radiosensitive. Data represents mean ± SEM of *n* = 5–8 mice per treatment group. * denotes *p* ≤ 0.05, NS denotes non-significant, as determined by Student’s *t*-test or log Mantel-cox tests on Kaplan-Meier plots.

RT is a radical primary treatment administered to patients with high-risk localised PCa, and also to patients with metastatic PCa. Therefore, therapeutic response to RT was evaluated in the DVL3 model and compared to the TRAMP C1 model. Established tumours (100–200 mm^3^) were assigned to treatment groups. The mice received either a single high dose of 8 Gy, which is often prescribed to treat PCa patients with metastatic disease; or conventional fractionated RT of 2 Gy administered over 5-consecutive days, which is similar to that used to treat localised disease (Figure 3A). Both single high dose (8 Gy) and fractioned RT (5 × 2 Gy) exhibited a growth delay compared to non-treated controls (0 Gy, *p* = 0.02, Figure 3B) and this improved survival of mice with engrafted DVL3 tumour cells (*p* = 0.03 and 0.02 respectively, Figure 3C). Conversely, neither the TRAMP C1 tumour size nor survival was significantly affected by either RT regime (Figure 3D,E). This demonstrates DVL3 allograft tumours are responsive to RT, including fractionated RT, whilst TRAMP C1 do not, as previously described (summarized in Figure 3F) [20].

### 2.4. Fractionated Radiotherapy Differentially Upregulated Genes Associated with STING/ Type-1 Interferon Signalling and Myeloid Signatures 

**Figure 4:**
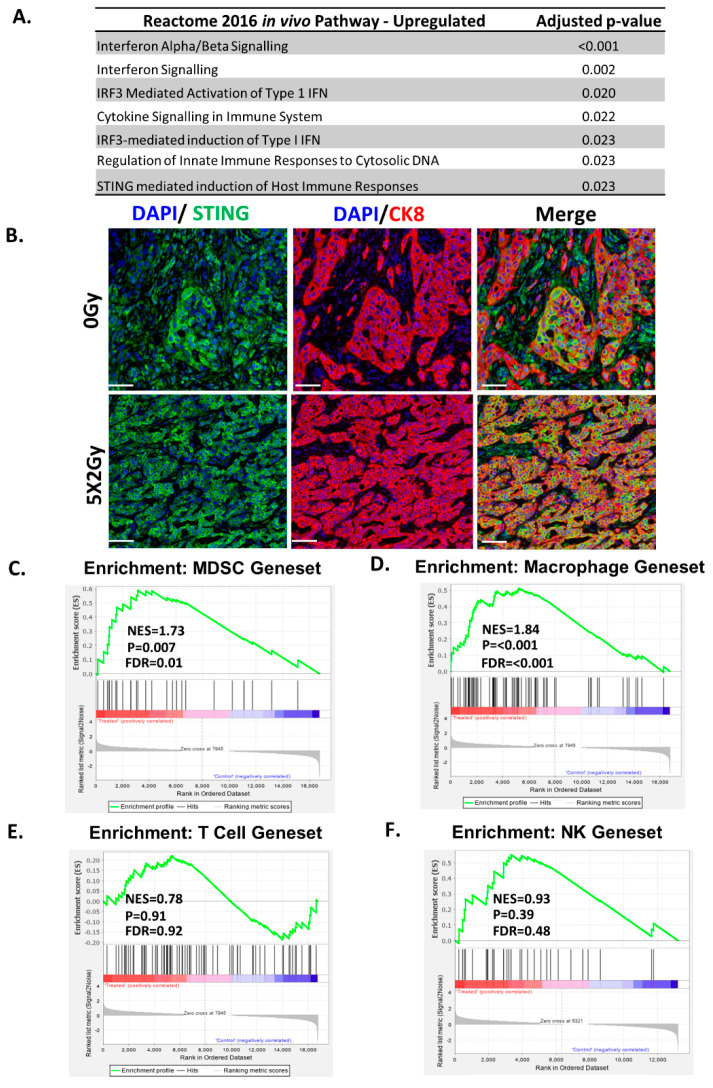
DVL3 immunosuppressive microenvironment enhanced by radiotherapy treatment. (**A**) Pathway enrichment analysis was performed using the online software Enricher for differential gene expression comparing the fractionated (5 × 2 Gy) syngeneic tumours to non-irradiated control (*n* = 3 samples per group). Upregulated pathways enrichment in the Reactome 2016 for transcripts with a significance of greater than 0.05 and fold increase of ≥1. Upregulated pathways include interferon signalling and immune response. (**B**) In keeping with RNA sequencing analysis, STING immunohistochemical analysis (Green) revealed a significant upregulation of expression in response to RT primarily within glandular epithelium as demonstrated via co-localisation with CK8 (Red). DAPI nuclear counterstain shown in blue. Scale bar equals 50 μm. Image representative of *n* = 3 independent tumour samples. GSEA enrichment analysis of tumours treated with fractionated (5 × 2 Gy) radiotherapy demonstrates an upregulation of (**C**) MDSC and (**D**) macrophage and signatures, but no enrichment of (**E**) T-cell or (**F**) NK cell signatures when compared with non-irradiated tumours. NES: normalized enrichment score, FDR: false discovery rate.

RT induces immunological changes in tumour cells and can re-calibrate the immune contexture of the tumour microenvironment [21]. In order to establish how fractionated (5 × 2 Gy) RT altered the tumour immune microenvironment, RNA sequencing analysis was performed on DVL3 tumours excised a week after final dose of RT (Figure 3A). Interestingly, ENRICHR pathway analysis on differentially expressed genes highlighted an enrichment for pathways involved in interferon alpha/ beta (IFN-b) signalling (*p* < 0.001), IRF3 mediated activation of type-1 Interferon (IFN) pathway (*p* = 0.02), regulation of innate immune responses to cystolic DNA (*p* < 0.05), and stimulator of interferon genes (STING) mediated immune response (*p* < 0.05, Figure 4A). RT also led to significant downregulation of pathways involved in extra cellular matrix remodelling, collagen degradation and activation of matrix metalloproteinases (Supplementary Figure S7A).

In order to establish phenotypic significance of the activation of type-1 IFN-pathway, the expression of STING was evaluated via immunohistochemistry in the irradiated DVL3 tumours. Fractionated RT led to a substantial, but non-significant increase in STING expression compared to non-treated controls (Supplementary Figure S7B). The increase in STING expression was primarily expressed by tumour epithelium in glandular structures. This was confirmed by multiplex immunohistochemistry, demonstrating STING co-localisation primarily in CK8+ tumour cells (Figure 4B, Supplementary Figure S8). Although STING expression was highest in tumour epithelium, this was not exclusive, as STING was also observed in the stromal and other cellular compartments (defined here as CK8 negative areas). Taken together with the RNA seq analysis, our data indicates towards a global upregulation of STING, and associated pathways within the tumour.

STING-dependent cytosolic DNA sensing promotes type-1 interferon response which is critical for activation and influx of MDSC population after radiation [22], and it is well documented that it can also enhance both T-and NK cell activation [23,24]. Therefore, Gene Set Enrichment Analysis (GSEA) was performed using the published gene set for MDSCs signature [25]. In the irradiated DVL3 tumours there was enrichment of the MDSC gene signature (Figure 4C; *p* = 0.007). We also performed GSEA analysis for gene signatures associated with activated macrophages, T-cells and NK cells, however, interestingly our data showed only upregulation of transcripts associated with activated macrophages (Figure 4D; *p* < 0.001), but not the cytotoxic T-cells (CD8) nor the natural killer T-cells or any other associated pathways (Figure 4E,F).

### 2.5. Fractionated Radiotherapy Resulted in an Increase in Infiltration of Myeloid Cells, but no Changes Were Observed in T-Cell Infiltrates 

**Figure 5:**
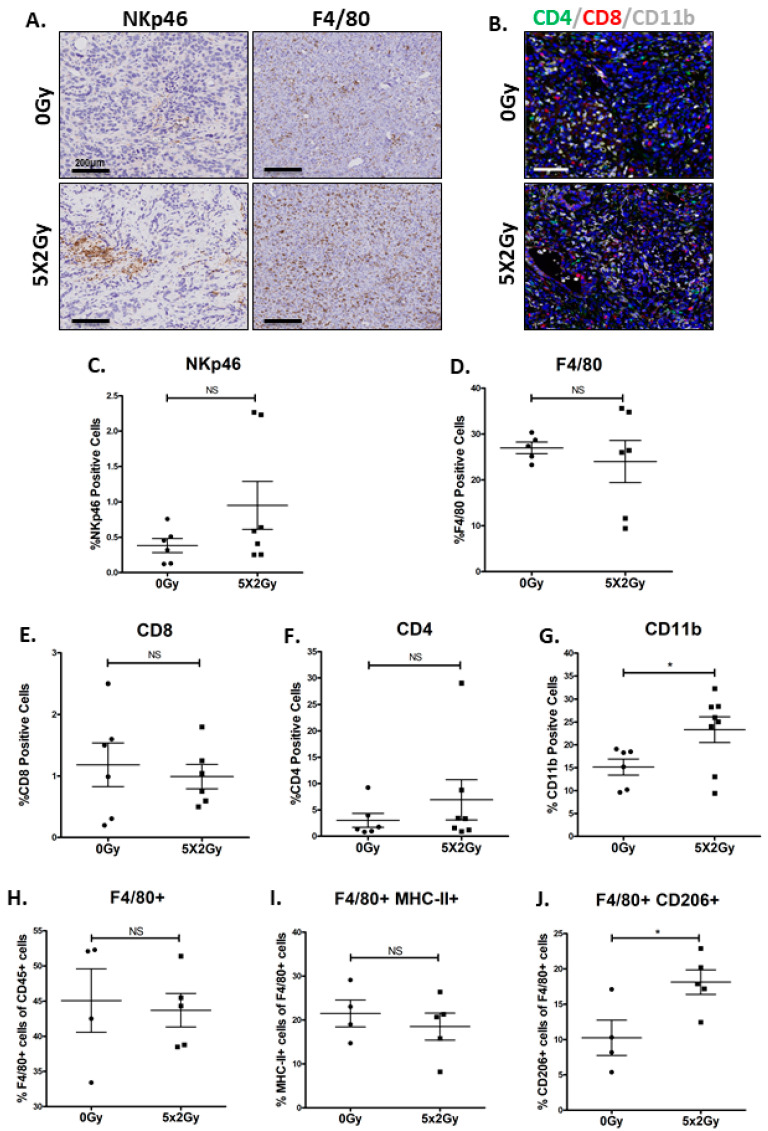
Radiotherapy resulted in an increase in immunosuppressive myeloid cells infiltrates and macrophages acquiring an M2 phenotype in the DVL3 tumours. (**A**) Representative immunohistochemical staining for natural killer T-cells (Nkp46) and macrophages (F/480) in DVL3 tumours. Nkp46 staining was sparse and mainly localised around the necrotic areas within the tumour. (**B**). Representative multiplex staining images for CD4 (green), CD8 (red) and CD11b (white) in the DVL3 tumours revealing an increase in CD11b staining in the irradiated tumours. Quantification of immunohistochemical staining comparing untreated (0 Gy) and tumours treated with fractionated radiotherapy (5 × 2 Gy) showed no significant difference in (**C**) NK-T-cells (NKp46), (**D**) macrophages (F4/80), (**E**) cytotoxic T cells (CD8), or (**F**) helper T cells (CD4). (**G**) However, radiotherapy treatment did induce a significant increase in myeloid cells (CD11b+) in the irradiated tumour. (**H**) Flow cytometry analysis for macrophages (gated as CD45+/F480+) cells showing no significant increase in proportion of cells in the irradiated tumours. (**I**) Flow cytometry analysis for MHC-II+ cells (M1 marker) on macrophages gated as (CD45+/F480+/ MHC-II+) in the irradiated tumours (**J**) Flow cytometry analysis for CD206 (Mannose receptor, an M2 marker) on macrophages gated as (CD45+/F480+/CD206+) revealing a significant increase in CD206+ staining in the irradiated tumours (gating strategy in Supplementary Figure S5). Data represents mean ± SEM of *n* = 4–8 independent murine tumors as indicated by points on each graph. * denotes *p* ≤ 0.05, NS denotes non-significant, as determined by Student’s *t*-test (immunohistochemistry) or Mann-Whitney, nonparametric testing (flow cytometry).

In order to establish how RT induces phenotypic changes in the immune cell infiltrates in our novel syngeneic tumour model, immunohistochemical staining was performed for T cells and myeloid cells. A marginal increase in natural killer T cells was observed in a portion of irradiated tumours, which predominantly localised around the necrotic areas, defined by morphological assessment and cleaved caspase-3 staining (NKp46, Figure 5A,C, Supplementary Figure S9A). Fractionated RT did not result in a significant change to the macrophage population (F4/80, Figure 5A,D). 

Using multiplex immunohistochemistry, T-cell (CD4, CD8) and myeloid cell (CD11b) expression were investigated in excised tumours (Figure 5B). RT did not result in an increase in either CD8+ or CD4+ T cells (Figure 5E,F). However, RT lead to an increase in myeloid cell infiltration (CD11b+) in the irradiated tumours compared to non-treated tumours (23.32% vs. 15.15%, *p* = 0.04. Figure 5G). As the F4/80 macrophage numbers showed no difference after administration of fractionated RT, the increase in CD11b myeloid cells could be due to an increase in either granulocytic and/or monocytic MDSCs.

Next, the expression of program cell-death ligand 1 (PD-L1) was investigated in the irradiated DVL3 tumours [26]. We have previously demonstrated that IFN-ƴ produced by CD8+ T-cells can cause adaptive upregulation of PD-L1 on tumour cells after delivery of low dose fractionated radiotherapy [27]. RT can also lead to increase in expression of PD-L1 on MDSCs and macrophages as demonstrated previously in a range of tumour models at higher doses [24]. Interestingly, in the DVL3 tumours, fractionated RT had no impact on PD-L1 expression measured as median fluorescence intensity on the surface of tumour cells, MDSCs cell or the macrophages (Supplementary Figure S9B–D).

We also investigated whether RT had an impact on macrophage reprograming from M2 to M1 phenotype. Using flow cytometric analysis, we demonstrate that although there was no significant difference in either the macrophage numbers (Figure 5H) or in the proportion of macrophages expressing MHC-II (an M1 marker) in the irradiated tumour (Figure 5I). However, the macrophages were skewed towards an immunosuppressive M2 phenotype, defined as (F480+/CD206+) cells (*p* = 0.03; Figure 5J). These findings taken together with RNA sequencing data suggest that the activation of type-1 signaling in the irradiated DVL3 tumours could be due to influx of MDSCs and activation of myeloid innate immune response pathway, and not driven by T cells.

## 3. Discussion

Whilst significant progress has been made in the treatment of metastatic, castration-resistant prostate cancer (CRPC), the majority of these cancers remain incurable. In order to have greater impact, it is important to understand the involvement of the TME in PCa evolution and treatment resistance, so that more effective treatment combinations can be developed for molecular high risk disease. RT remains an important treatment for primary localized PCa, and is increasingly used for treatment for metastatic disease [28,29,30,31,32]. Although both *Pten* and *p53* loss have been implicated in treatment failure, the narrow range of PCa pre-clinical models limits therapeutic development. This study has developed two novel murine cell models; mPECs, which model normal prostate epithelium and the tumorigenic DVL3 cell line derived with genetically relevant drivers (*Pten^−/−^/trp53^−/−^)* which has been extensively characterized for sensitivity to RT and TME response. Additionally, the mPEC model serves as a normal epithelial control, but also provides a key model for further genetic manipulation to examine pathways thought to be important in tumour development or immune response in a syngeneic environment. 

Transgenic tumours from which the DVL3 cells were generated, were derived via deletion of *Pten* and *trp53,* genes that are frequently mutated in human PCa and are implicated in aggressive forms of the disease [10]; thus, avoiding the introduction of tumorigenic viral proteins such as SV40 large T-antigen, which was used to generate the TRAMP C1 cell line [6]. Although the TRAMP C1 cell line has contributed significantly to PCa research, recent evidence indicates it generates neuroendocrine tumours, which clinically equates to only 0.5–2% of all PCa cases [7]. Furthermore, although other syngeneic PCa models exist [33,34] (e.g. MyC-CaP and Pten-CaP8), they have not been well characterized for their similarity to human disease, and immune microenvironment. The DVL3 cell line develops tumours that represent an adenocarcinoma phenotype much more akin to the majority of human PCa, and displays a similar TME to patient disease. The DVL3 model also sets itself apart, as it can be easily generated via subcutaneous allograft into immune-competent mice. As such, not only is it both time and cost beneficial compared with conventional GEM models, the tumours are easily accessible for therapeutic intervention, such as local delivery of RT and tumour assessment.

Transgenic models of PCa, and subsequent cell lines, generated from the *Pten^−/−^/trp53^−/−^* model generate heterogeneous tumours, forming distinct glandular structures [10]. Arguably, heterogeneity is one of the greatest benefits of this model, as most cell-based models are thought to be clonally selected due to genetic drift. As PCa is a multifocal heterogeneous disease, the derivation of a bulk DVL3 population allows for research into PCa evolution and response to treatment [35]. Tumours formed by the DVL3 model maintained the heterogeneous CK8+/CK5+ glandular structures and regions of AR positivity, similar to the tumours from which they were derived. Additionally, unlike other *Pten^−/−^/trp53^−/−^* murine cell lines, previously described in the literature, the DVL3 cells can be implanted in immune competent C57BL/6 mice, presenting with immunological cold tumour immune microenvironment, mirroring observations seen clinically [10,36].

Development of immune therapies as a method of harnessing the immune system’s anti-tumour effects has recently gained traction [36]. Immune checkpoint inhibitors (ICI) have been used successfully to treat melanoma and lung cancers; however, their impact on PCa is more limited [37]. Unlike melanoma and lung cancer, PCa has low levels of infiltrating CD8+ T cells, high proportion of MDSCs and tumour promoting M2 macrophages, therefore ICI are unlikely to work in majority of PCa patients [36,37]. Furthermore, variations in the PCa tumour microenvironment have been reported to correlate with PTEN-loss, defects in DNA repair pathways, low levels of tumour-associated antigens and differences in the immune function of patients [37]. PTEN loss occurs in 20% of primary PCa patients and has been associated with poorer overall survival [9,10,37]. Additionally, melanoma patients with PTEN loss also have lower levels of tumour infiltrating lymphocytes (TIL) [37]. We have confirmed that this is also true in the tumours resulting from DVL3 syngeneic engraftment, underscoring the more immuno-suppressed microenvironment of these tumours compared to the TRAMP C1 tumours. 

Comparing the DVL3 allografts to published transgenic models driven by *Pten* deletion highlights other similarities and opportunities for future studies. For example *Pten* deletion is known to lead to the expansion and immunosuppressive activities of Gr1^+^/CD11b^+^ myeloid derived suppressor cells (MDSC) [13]. High numbers of MDSCs in a *Pten*^−/−^ setting provide a protective effect on PCa cells from senescence, therefore sustaining tumour growth [13,38]. Mechanistically this has been observed to occur in CRPC patients and in transgenic prostate cancer models through the activation of AR signalling due to paracrine IL-23 secretion by MDSCs [39]. Furthermore, influx of tumour MDSCs has been detected following RT, an observation which has been previously associated with the RT-induced up-regulation of STING expression in tumour cells [22,40,41]. Targeting MDSC, through IL-23 inhibition, in combination with RT and ADT could therefore provide a survival benefit for PCa patients. 

STING mediated type-1 IFN production can be an effective approach to cancer therapy, due to its role in T-cell priming and dendritic cell activation. Counterintuitively, STING activation can also be immunosuppressive as previously reported [24]. In the DVL3 model, RT mediated STING activation does not result in enrichment of T- or NK cell gene signatures, nor did it result in a significant increase in the proportion of T- or NK cells, suggesting dominant immunosuppressive effects in the TME. However, we did note aggregation of NK-cells in and around necrotic areas after RT [42]. Further studies using the DVL3 model are necessary to clarify the role of NK-cell mediated cell killing, which may have potential therapeutic implications. Interestingly, in spite of the IFN-type-1 pathway activation, no significant change in PD-L1 expression was observed either in the tumor or macrophages after RT. It has been demonstrated that the kinetics of PD-L1 expression can be transient, time, and radiation dose-dependent [27,43]. In this study, we investigated the expression of PD-L1 at a single time point (a week after RT), and after administration of low dose fractionated RT (5 × 2 Gy). As such, further investigations are required with high radiation dose and observation and differing time points to establish the kinetics of PD-L1 upregulation on tumour cells and macrophages. Furthermore, PD-L1 expression on MDSCs can be activated by type-1 IFN in an autocrine manner, which is partially controlled by IFNAR1 expression on MDSCs [44]. Our gene expression analysis demonstrated no significant change in the expression of IFNAR1 with RT (data not shown). This may partly explain our findings, however, we cannot fully rule out other environmental factors. 

Overall, the DVL3 model recapitulates clinically relevant immune microenvironment features that have been previously observed in PCa transgenic models following RT or androgen deprivation therapy, such as influx of MDSC, M2 tumour promoting macrophages, and lack of cytotoxic T-cell priming [13,39,41]. Although similar models have previously been published upon [10,45,46], to our knowledge this is the first report of a model which has been proven to be syngeneically engraftable and is driven by relevant genetic drivers to prostate cancer (*Pten* and *trp53*), and mirrors clinical response. The genetic status of the model, resultant histological Gleason score, and immunological features suggest the DVL3 model is currently poised for extensive future work as a model of molecular high-risk localized disease, which is benefited by its cost and time effectiveness compared with other models, ease of accessibility and tumour tracking, and its accurate recapitulation of PCa patient disease. However, further characterization of the model is required to examine if it is also suitable as a model of metastatic disease via orthotopic engraftment to the prostate or bone. 

## 4. Materials and Methods

### 4.1. Cell Line Derivation and Maintenance 

Mouse prostate epithelial cells (mPEC) were generated from normal dorsal, ventral and lateral lobes of the prostate from Probasin Cre^−/−^ (Pb-Cre4) mice. Murine prostate cancer cells (DVL3) were generated from tumours derived from the dorsal, ventral and lateral prostate lobes of a *Pten^−/−^/trp53^−/−^* Pb-Cre4 mouse [10]. These lobes were specifically utilised for both models as they are morphologically more similar to the peripheral zone in the human prostate gland which generates adenocarcinoma [11] and these lobes are also those which generate adenocarcinoma in the *Pten^−/−^/trp53^−/−^* Pb-Cre4 murine model. mPEC and DVL3 cell line generation is summarised in Supplemental Figure S1. Briefly, tissue fragments were collected in PBS and manually dissociated into 1 mm^3^ fragments using a scalpel. The sample was centrifuged at 2000 rpm to remove extraneous blood and fat, and remaining tissue digested in 1 mg/mL collagenase/dispase in PBS for 30 min at 37 °C, after digestion the tissue was further disrupted via serological pippetting. The supernatant containing prostate cells was collected, and the collagnease/dispase inactivated with EDTA. The digestion process was repeated twice with remaining tissue fragments. Supernatant from the digestions was pooled and centrifuged at 2000 rpm and prostate cells were re-suspended in RPMI supplemented with 10% FBS, 1× penicillin/streptomycin, 100 nM DHT and 10 μmol/L ROCK inhibitor (Y-27632, Sigma, Gillingham, UK). These cells were then incubated for 10 min at 37 °C to allow for prostate fibroblasts to adhere to the tissue culture plastic. Remaining cell suspension, enriched for prostate epithelium was transferred to a new culture flask. This process of differential plating, allowing for initial adherence of fibroblasts and removal and retention of less adherent epithelial cells, was repeated for 10 passages, at which time use of the ROCK inhibitor (Y-27632, Sigma) was stopped and the population was characterised as epithelial. After isolation and initial 10 passages to reach a homogenous epithelial population, the mPEC and DVL3 cell lines were maintained in RPMI-media supplemented with 10% FBS, L-glutamine and 100 nM DHT.

The TRAMP C1 murine prostate carcinoma cells were purchased from ATCC (Manassas, VA, USA) and maintained in DMEM high glucose medium, supplemented with 4 mM L-glutamine, 5% FBS, 5% Nu Serum (Corning, Bedford, MA, USA), 0.005 mg/mL of bovine insulin, and 10 nM dehydroisoandrosterone (Sigma). CT26 murine colon carcinoma cells (ATCC) and 4434 cells isolated from BRAFV600E p16-/- mice (Richard Marias, Cancer Research UK Manchester Institute, Manchester, UK) were maintained in DMEM, and 4T1 triple-negative breast cancers (ATCC) maintained in RPMI-media supplemented with 10% FCS, 1% L-glutamine (Invitrogen, Paisley, UK).

### 4.2. Syngeneic Modelling

C57BL/6 and BALB/C male mice (8-weeks old) were obtained from Harlan (Derby, UK). All animal experiments were performed under United Kingdom Home Office Licenses held at Queen’s University Belfast or the CRUK Manchester Institute, University of Manchester (PPL2775; PCC943F76 respectively). Prior to each in vivo experiment, cells were screened for mycoplasma contamination and mouse hepatitis virus (MHV). Mice were housed on a 12/12 light/dark cycle and were given filtered water and fed *ad libitum*. C57BL/6 male mice were inoculated subcutaneously with either 5 × 10^6^ TRAMP C1, 1 × 10^6^ DVL3 cells, 1 × 10^6^ mPEC cells or 5 × 10^6^ 4434 cells; BALB/C mice were inoculated with 5 × 10^5^ CT26 cells, 1 × 10^5^ 4T1 cells under light general anaesthetic using Isoflurane and oxygen gaseous mix in accordance with project license and home office regulations. Tumour volume was measured using calipers as length × (width)^2^/2. 

### 4.3. Sample Preparation 

Tumour bearing mice were sacrificed at the indicated time points using standard schedule-1 procedure, and in accordance with current UK home office legislation. Tumour samples were cut into half and either fixed in 4% buffered formalin (Sigma Aldrich) for 24 h or collected in media for tumour disaggregation. The formalin fixed tumour samples were transferred to 70% ethanol and processed to FFPE blocks at the CRUK Manchester histology core facility. H&E slides obtained were evaluated by a dedicated uropathologist (PO) with the aim to compare with morphological features of human prostate cancer as well as estimate a Gleason pattern and score.

### 4.4. Immunohistochemistry

Briefly, the slides were deparaffinised in xylene followed by rehydration in ethanol. Antigen retrieval was performed using pH 6 citrate buffer, followed by 3% H_2_O_2_ block. Slides were incubated with 10% serum, prior to incubation with primary antibody overnight at 4° (Table S1). Primary antibody was detected with either HRP detection kit or biotinylated secondary antibody followed by ABC detection kit (Vector Labs, Burlingame, CA, USA). Slides were briefly incubated in DAB substrate (Vector Labs), washed in water, and counter-stained using haematoxylin. STING and cleaved Caspase-3 staining was performed on the BOND Rx automation system (Leica, Microsystems, Milton Keynes, UK) using standard protocol. For multiplex staining of mouse FFPE tumour sections, the opal TSA detection system (Opal 520^®^, 570^®^ and 650^®^) were applied to sections following manufacturer’s instruction and run on Leica BOND Rx automated system. The slides were counterstained with DAPI. Chromagen slides were scanned digitally using Leica SCN 4000 slide scanner (Leica Microsystems) VS-200 slide scanner (Olympus, Essex, UK), and the multiplex slides were scanned using Versa Slide scanner (Leica Microsystems) and VS-120 slide scanner (Olympus). Image analysis and quantification was performed using Definiens^®^ Tissue Phenomics Software (Munich, Germany) or Halo^®^ image analysis (Indica Labs, Albuquerque, NM, USA). Quantification of the percentage of positive cells was determined using either hematoxylin or DAPI staining to identify total cell count. Scoring of positive staining of chromogen slides (summarized in Figure 1C) was performed in a blinded fashion by two independent researchers. For quantification of co-localisation using Halo^®^; High plex FL Ver 3.1.0 module was used. 

### 4.5. Immunocytochemistry

Cells were seeded onto glass slides pre-coated with collagen type II (BD Biosciences, San Jose, CA, USA) and left to adhere for 24 h before fixation with 4% paraformaldehyde at RT. Cells were permeabilised with 0.1% Triton X and blocked with 3% FBS-PBS. Primary antibody was added to slides and incubated for 1 h at RT followed by incubation in secondary antibody for 1 h at RT (Table S1). Coverslips were mounted onto glass slides using Mounting media (Invitrogen) containing Dapi and imaged.

### 4.6. RNA Isolation and cDNA Synthesis 

Total RNA from cells was isolated using TriPure Isolation reagent (Roche, Mannheim, Germany) and phenol-chloroform extraction and cDNA synthesised using first strand cDNA (Roche) according to the respective manufacturer’s instructions.

### 4.7. qRT-PCR

mRNA levels were determined by RT-PCR and measured on Roche LightCycler^®^480 II with SYBR Green I Master (Roche). All primers were obtained from Eurofins (Ebersberg, Germany) and sequences are detailed in (Table S2). mRNA expression was evaluated using β-actin and GAPDH housekeeping genes and is shown normalized to β-actin as a fold change relative to mPECs transcript levels.

### 4.8. Western Blotting

Cells were lysed with RIPA buffer (Sigma) supplemented with Protease Inhibitor Cocktail (Roche), centrifuged and supernatant containing protein analysed by SDS Page (Invitrogen). Proteins were transferred onto a nitrocellulose membrane, blocked for 1 h in 5% milk TBS-T prior to incubation with primary antibody at 4 °C overnight, concentrations are detailed in (Table S2). Complementary secondary antibodies were used at 1:3000. Membranes were visualised via chemiluminescence on a Syngene G:Box imager using reagent (Merck, Cambridge, UK).

### 4.9. Flow Cytometry on Cell Lines

Cells were incubated with primary antibody conjugated fluorophore for 30 min at 4 °C, washed with PBS and analysed on the Introducing the BD Accuri™ C6 Plus according to the manufacturer’s instructions.

### 4.10. Radiotherapy

Upon tumour establishment (4–6 weeks post inoculation), mice were randomised to treatment groups. Irradiation was performed when the tumours were between (100–200 mm^3^). The tumour bearing mice were placed in a lead jig with an opening for the tumour and shielding for the rest of the body. Radiotherapy was delivered using an AGO cell X-Ray unit and XSTRAHL (CIX3) in vivo irradiator at a dose rate of 2.086 Gy/min. Tumour bearing mice received either single dose of 8 Gy irradiation, or 5 fractions of 2 Gy delivered over 5 consecutive days (as shown in Figure 3A).

### 4.11. RNA Extraction for RNA Seq Analysis

RNA from FFPE mouse tumour tissue was extracted. Briefly, 2–4 (10 μM) sections were transferred into RNAase free microcentrifuge tube. The paraffin was removed by incubating in xylene and ethanol. For lysate and total RNA purification, digestion buffer and proteinase-K was added to the samples as per manufacturer’s instructions (Norgen Kit, Ontario, Canada). The samples were spun briefly followed by transferring the supernatant to a new RNAase free microcentrifuge tube. The RNA containing tubes were incubated for 15 min at 80 °C. The lysates were then passed through RNA purification microcolumn and centrifuged for 1 min at 14,000 RPM. The microcolumns were washed according to manufacturer’s instructions and the RNA eluted using the Elution solution (Norgen Kit).

### 4.12. RNA Library Preparation, Sequencing and Analysis 

RNA libraries preparations were generated using the Quant-seq 3’ mRNA-Seq FWD kit (PART NO k15.96, Lexogen, Vienna, Austria) according to manufacturer’s instructions, with 500ng input RNA. FFPE-derived RNA was prepared using the suggested manufacturer’s instructions. Libraries were pooled and sequenced on the NextSeq 500 using the Queen’s University Genomics Core Technology Unit, yielding an average of 10 million reads per sample. FASTQ files were aligned to the mm10 genomic reference using STAR aligner [47] and counts quantified at a gene level with HTseq-Counts [48] to yield 5-8M mapped reads per sample. Differential gene expression was performed between the irradiated and un-irradiated samples using the DESEQ 2. Differentially expressed genes were filtered; FDR adjusted—*p* Value < 0.05 and Log2 Fold-Change ≥1 (up-regulated) or ≤−1 (down-regulated). Pathway analysis was performed on filtered genelists using the Reactome database [49] in Enrichr [50,51]. To identify enrichment of immune populations with the RT tumours vs untreated, enrichment analysis was performed using the open assess tool gene set enrichment analysis (GSEA) [52,53] with 1000 gene label permutations. Immune cell gene signatures for macrophages, NK cells, T-cells and MDSC were taken from previous published studies [25,54,55].

### 4.13. Flow Cytometry on Tumour Tissue

To obtain single cell suspensions, tumours were processed using a gentleMacs dissociator and a murine dissociation kit (Miltenyi Biotec, Surrey, UK). For staining of cells, non-specific binding was blocked with rat anti-CD16/CD32 Fc block on ice. Cells were incubated with Gr-1–FITC, CD11b–APC (eBioscience, Leicestershire, UK), washed in 1% FCS/PBS. For analysis, live cells were gated using vital dye exclusion (Invitrogen) and population phenotyped on FACs Canto (BD Bioscience) and analyzed using Flow Jo software. An example of the gating strategy employed is provided in Figure S5.

### 4.14. Statistical Analysis 

Results were analysed using GraphPad Prism (v7.03, San Diego, CA, USA). Data was analysed via a Shapiro-Wilk normality test was first used to confirm that groups were distributed normally. When comparing two groups, if the data was normally distributed it was analysed by Student’s *t*-test. Nonparametric data was analysed via Mann-Whitney testing. When comparing more than two groups a one-way analysis of variance (ANOVA) followed by a Tukey’s multiple comparisons test was used to detect significant differences between means. The limit for significance was set at *p* ≤ 0.05. Data are described with standard error of the mean (SEM). To compare survival curves from in vivo experiments, Log-Rank Mantel–Cox tests were performed on Kaplan–Meier plots.

## 5. Conclusions

This study highlights that the DVL3 model represents substantial progress in preclinical PCa modelling displaying similar molecular, histological, and microenvironmental features compared to alternative models. Furthermore, the DVL3 cells and subsequent tumours can be generated quickly and inexpensively compared to standard transgenic models, and tumours are readily accessible for therapeutic intervention. Importantly, DVL3 cells can be syngeneically engrafted into immune component hosts and respond to standard of care RT, with a similar immune influx, thus enabling future validation of novel therapeutics for PCa including immunomodulatory agents targeting MDSC, macrophages and agents that can prime T-cell responses either alone or in combination with standard of care RT or ADTs. 

## Supplementary Materials

The following are available online at https://www.mdpi.com/2072-6694/12/10/2804/s1, All antibodies utilised for immunohistochemistry, immunocytochemistry, western blotting and flow cytometry can be found in Supplementary Table S1. Further, all primers utilitsed for qRT-PCR can be found in Supplementary Table S2. RNA-seq files have been deposited on the Gene Expression Omnibus (GEO) database. GSE number—GSE146790. Figure S1: Summary schematic of DVL3 and mPEC cell model isolation, Figure S2: Further characterization of the DVL3 model compared with TRAMP C1 with focus on AR signaling, Figure S3: Additional high and low magnification immunohistochemical staining of DVL3 tumours, Figure S4: Whole blot images and densitometry for western blot experiments, Figure S5: Gating strategies for flow cytometry, Figure S6: Comparison of DVL3 to other commonly used syngeneic models, Figure S7: Reactome analysis of RNA sequencing revealing downregulated pathways and evidence of STING response, Figure S8: Additional STING analysis and quantification, Figure S9: Nkp46 expression around necrotic tissues and PDL1 expression, Table S1: List of antibodies utilised for immunohistochemistry, western blotting, immunocytochemistry and flow cytometry, Table S2: List of qRT-PCR primers.
