# Supplementary material for: Investigating Radiotherapy Response in a Novel Syngeneic Model of Prostate Cancer
Source: Cancers (Basel). 2020 Sep 29;12(10):2804. doi: 10.3390/cancers12102804 (PMC7599844; doi:10.3390/cancers12102804)
Supplement: Supplementary file 1 [file cancers-12-02804-s001.pdf]

cancers

MDPI

Supplementary Material: Investigating Radiotherapy Response in a Novel Syngeneic Model of Prostate Cancer

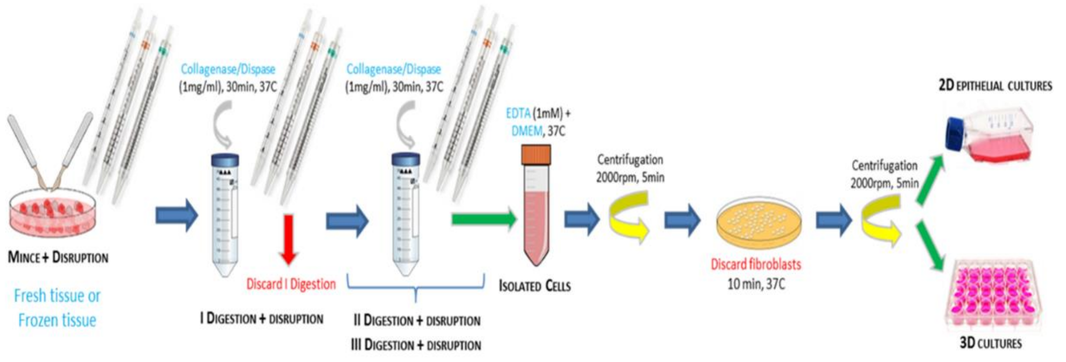

Figure S1. Summary schematic of DVL3 and mPEC cell model isolation from either tumour or normal prostate tissue respectively.

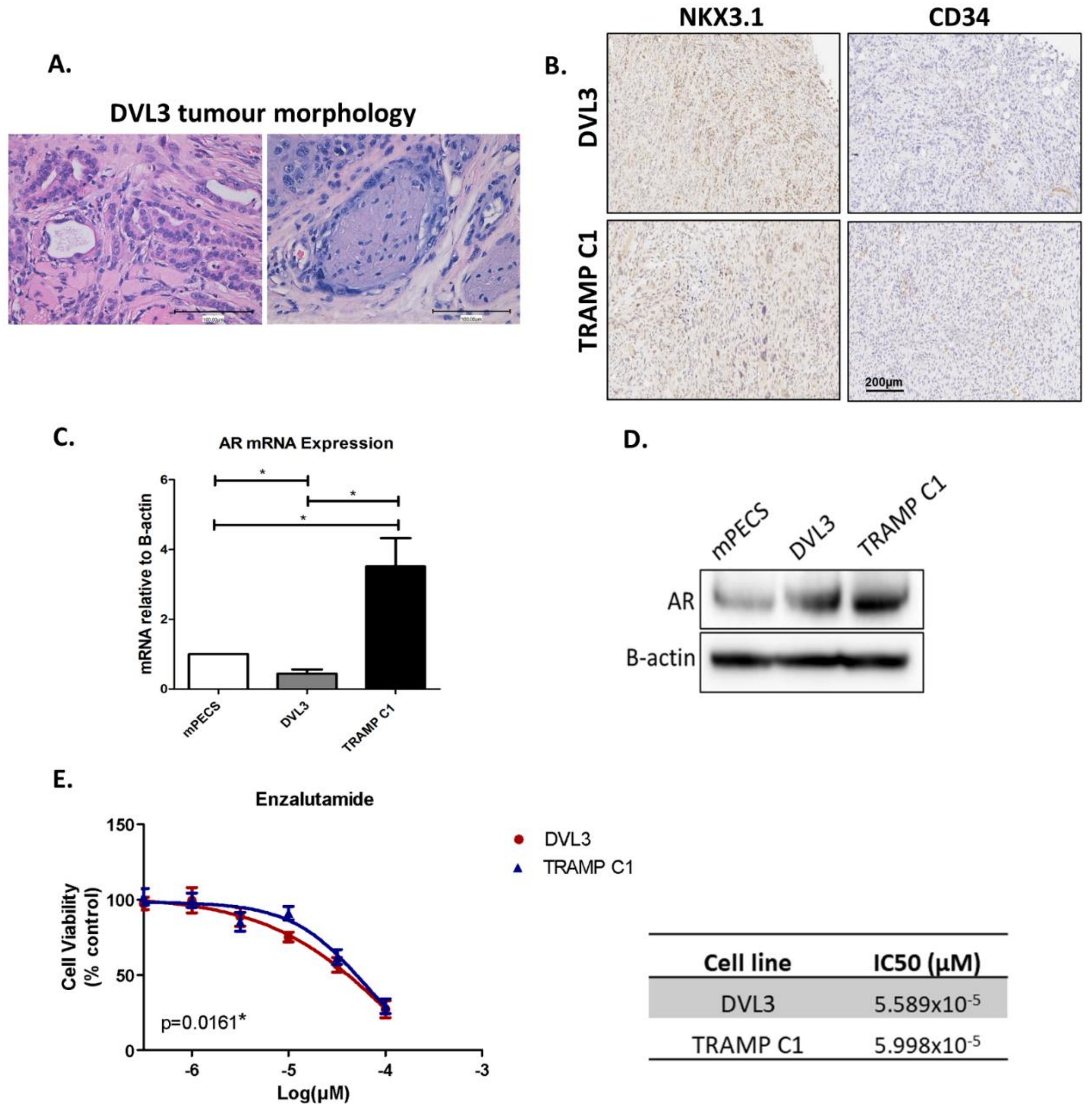

Figure S2. (**A**) DVL3 tumour morphology, demonstrating clear areas of glandular morphology similar to acinar adenocarcinoma with features in keeping with Gleason 7 (3 + 4); with focal perineural invasion, a common finding in human prostatic cancer. Scale bars equal to 100 μm. (**B**) Both DLV3 and TRAMP C1 expressed NKX3.1 and CD34 to a similar level as determined by immunohistochemistry. (**C**) qRT PCR revealed AR mRNA was significantly higher in TRAMP C1 cells. mRNA expression was normalised to β-actin expression and shown as a fold change relative to mPECs transcript levels. (**D**) AR protein (110 kDA) also appeared higher in the TRAMP C1 model compared to both the novel models (mPEC and DVL3) when analysed via western blot. (**E**) However, both DVL3 and TRAMP C1 were comparatively responsive to the androgen deprivation therapy Enzalutamide, with DVL3 cells appearing statistically more sensitive than TRAMP C1 cells. Data represents mean ± SEM of at least $n = 3$ replicates. * denotes $p \leq 0.05$, as determined by Student's t-test.

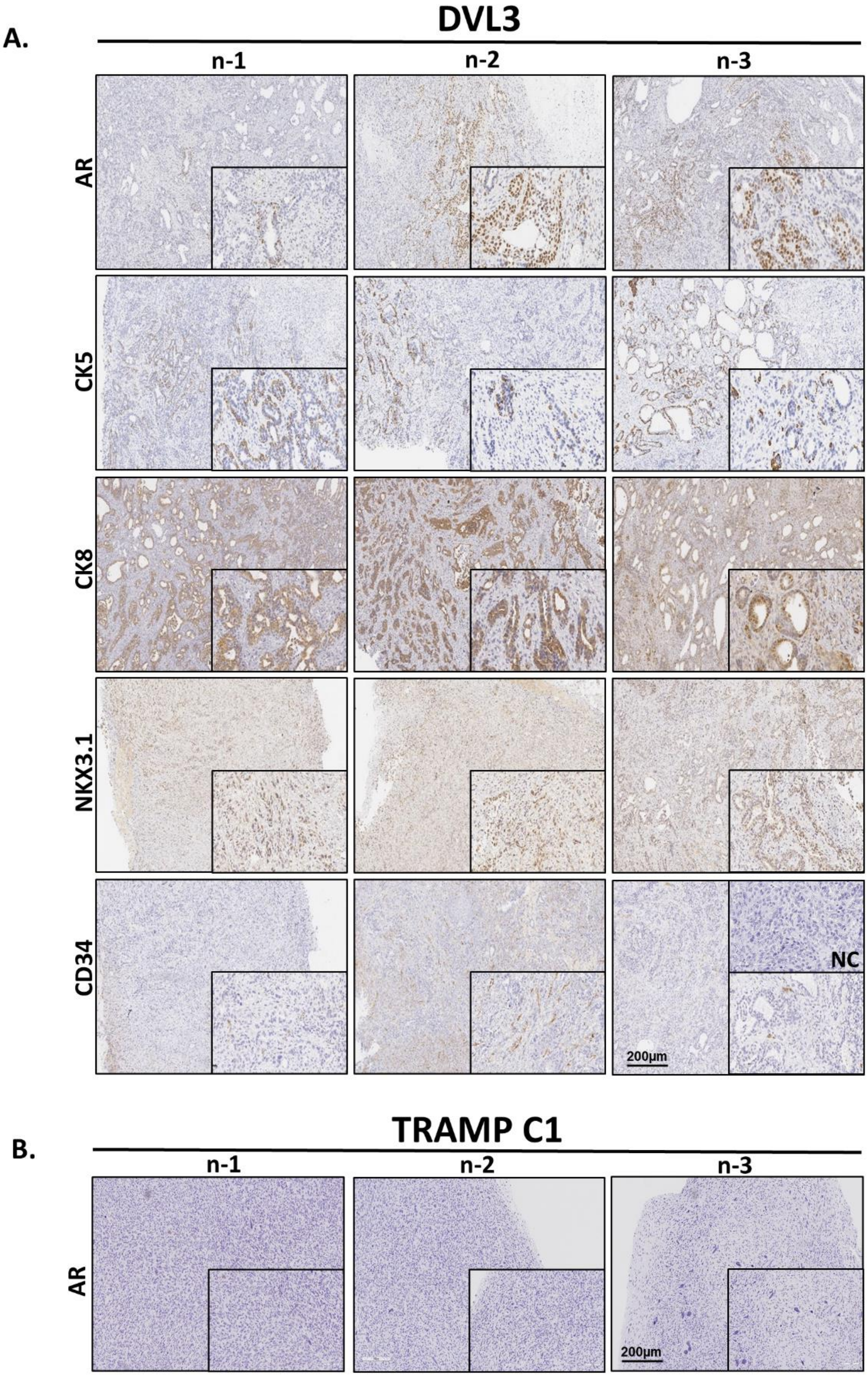

Figure S3. (**A**) DAB immunohistochmeical staining of $n = 3$ independent DVL3 tumours for androgen receptor (AR), cytokeratin 5 and 8 (CK5, CK8), NKX3.1, and CD34 (representative images in Figure 1(**B**) and summarised in 1(**C**) provided in both low and high magnification**.** NC denotes the inset of a no primary antibody negative control. (**B**) Representative AR staining of $n = 3$ TRAMP C1 tumours, denoting heterogenous staining - primarily absent expression in most tumours with some weak expression in the epithelium of other tumours. Scale bars equal 200µm.

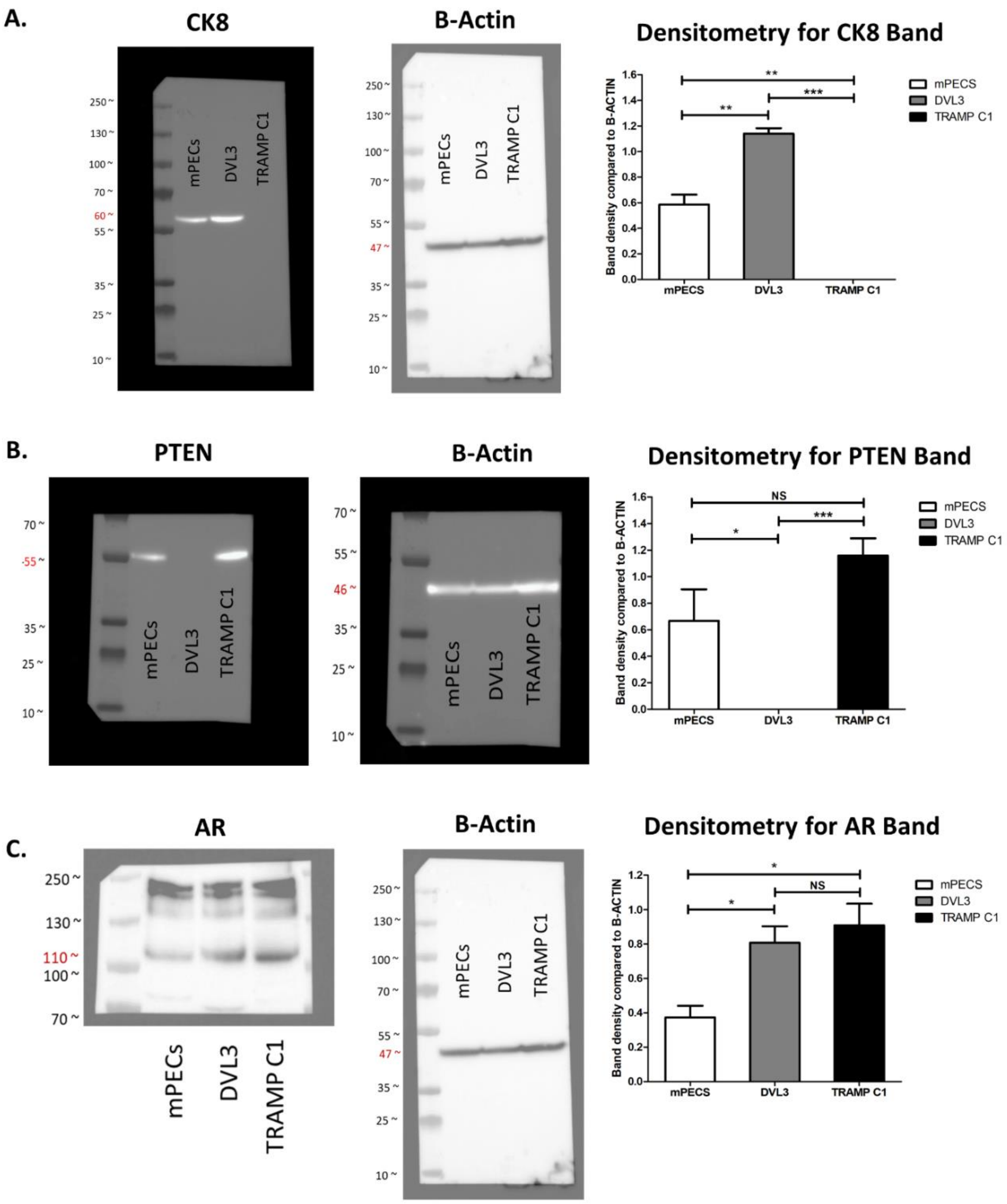

Figure S4. Representative whole blot western blotting images with densitometry from *n* = 3 independent experiments comparing (**A**) CK8, (**B**) PTEN and (**C**) AR expression to B-actin expression. Protein ladder molecular weights shown in black font with band of interest molecular weight denoted in red font. Data represents mean ± SEM. * denotes $p \leq 0.05$, ** denotes $p \leq 0.01$, *** denotes $p \leq 0.001$, NS denotes non-significant, as determined by Student's t-test.

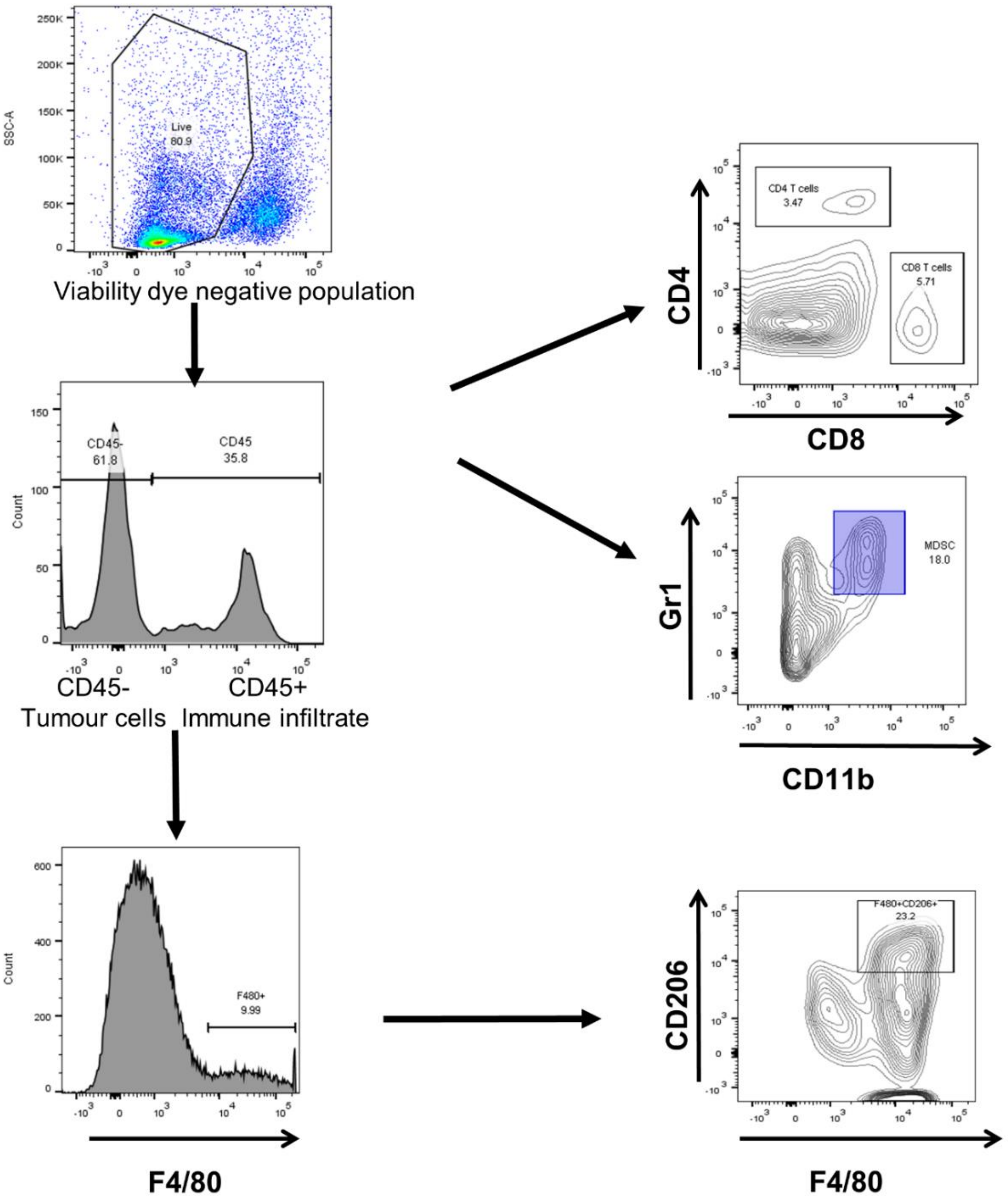

Figure S5. Example gating strategies for flow cytometry analysis for immune cells within the tumours. The live cells were gated as CD45 - (Tumour cells) or CD45+ (immune infiltrates) and subsequent gating applied on CD45+ cells for various immune markers. Helper T-cells were gated as either CD45+ CD4+ cells, whereas the cytotoxic T-cells were gated as CD45+ CD8+ cells. The MDSC were defined as dual CD11b+ and Gr1+ cells with the gating strategy applied on live CD45+ cells. Macrophage counts were obtained from the contour plots and defined as CD45+ F480+ cells. Further gating strategy was applied on F480+CD206+ cells which were classed as M2 macrophage marker.

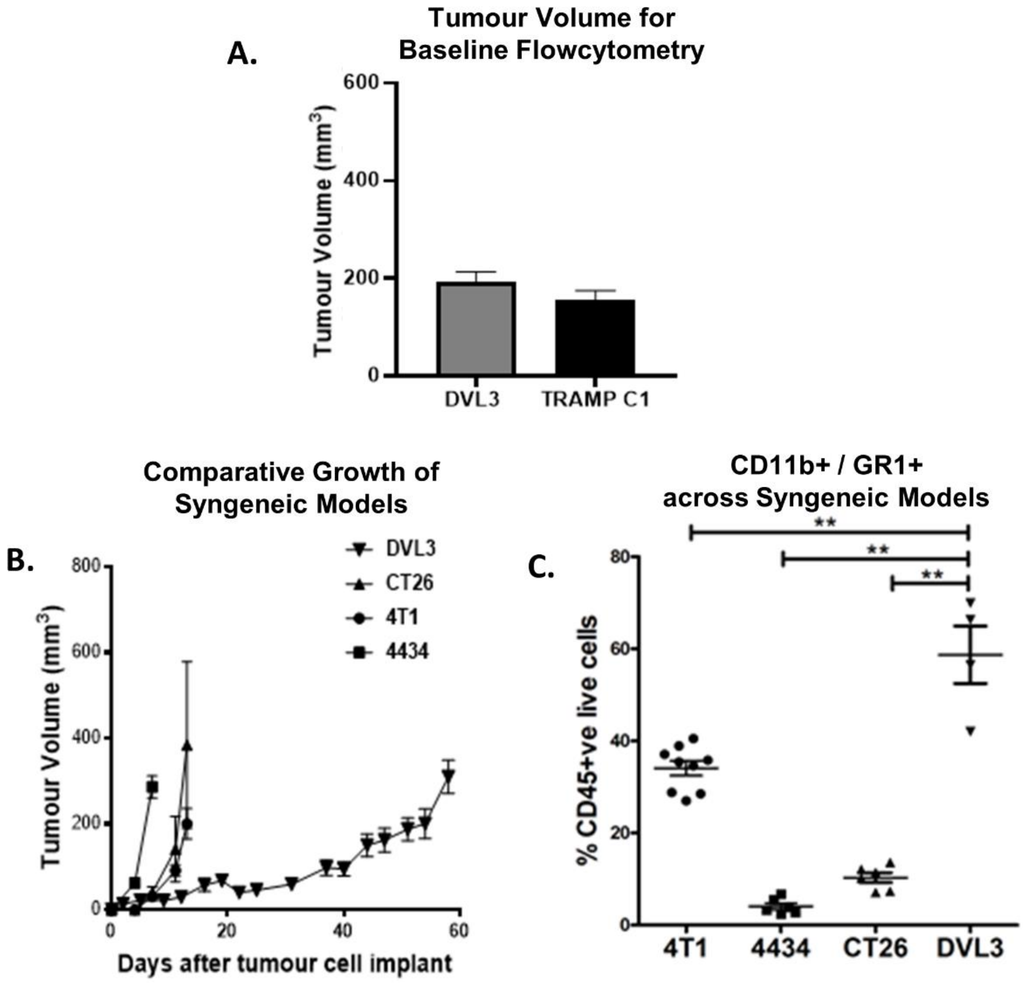

Figure S6. (**A**) Mean tumour volume of DVL3 and TRAMP C1 tumours for baseline tumour immune profiling performed in Figure 2. C57BL/6 male mice were inoculated with 1X10^{6} DVL3 or 5X10^{6} TRAMP C1 cells. Once the tumours were established ~200mm^{3}, tumours were excised and dissociated for flow cytormetric analysis of immune cells**.** (**B**) Representative tumour growth data for 4T1, 4434, CT26 and DVL3 tumour model showing difference in tumour growth rate. The DVL3 tumour models are slow growing tumours and have significantly reduced growth rate compared to other well established and characterised syngeneic murine models. Data represents mean tumour volume of (n = 4–7) mice per tumour lines. (**C**) The MDSC population in terminal endpoint DVL3 tumours when compared to other well characterised syngeneic tumour models breast (4T1), Melanoma (4434), and Colorectal (CT26), highlighting significantly higher proportion of CD11b+ GR1+ cells within the leukocyte compartment at any given time, and as the tumour develops. C57BL/6 mice were inoculated subcutaneously with either 1 x 10^{6} DVL3 cells, or 5 x 10^{6} 4434 cells; BALB/C mice were inoculated with 5 x 10^{5} CT26 cells, 1 x 10^{5} 4T1 cells. Tumours were excised and mechanically dissociated into a single cell suspension for flow cytometric analysis. This data along with the baseline characterisation (Figure 2) suggest that the tumour microenvironment of DVL3 tumours is highly immunosuppressive driven by myeloid cells.

A.

Reactome 2016 *in vivo* Pathway –Downregulated	Adjusted p-value
Extracellular Matrix Organization	<0.001
Elastic Fibre Formation	<0.001
Collagen Degradation	0.022
Molecules associated with Elastic Fibres	0.022
Activation of Matrix Metalloproteinases	0.022
ECM Proteoglycans	0.025
Degradation of the Extracellular Matrix	0.042

B.

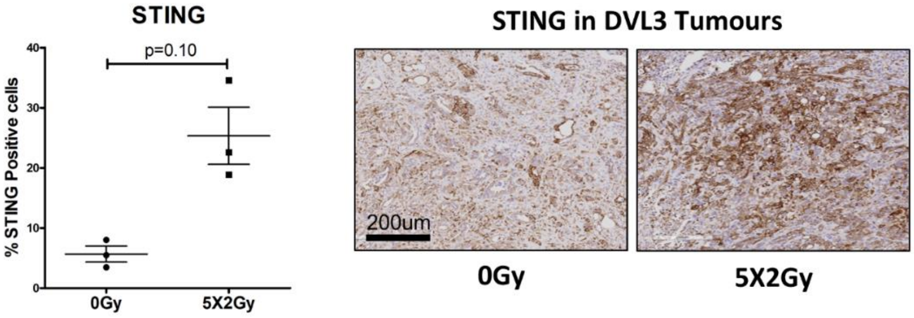

Figure S7. (**A**) Reactome analysis of RNA sequencing revealing downregulated pathways in DVL3 tumours post fractionated radiotherapy (RT, 5x2GY) compared to untreated tumours. Radiotherapy induces reduction of extracellular matrix organization, collagen degradation and activation of matrix metalloproteinases. Downregulated pathways enrichment in the Reactome 2016 for transcripts with a significance of greater than 0.05 and fold increase of ≥1. (**B**) Immunohistochemical analysis of STING expression demonstrates a trend toward increased STING protein in response to RT.

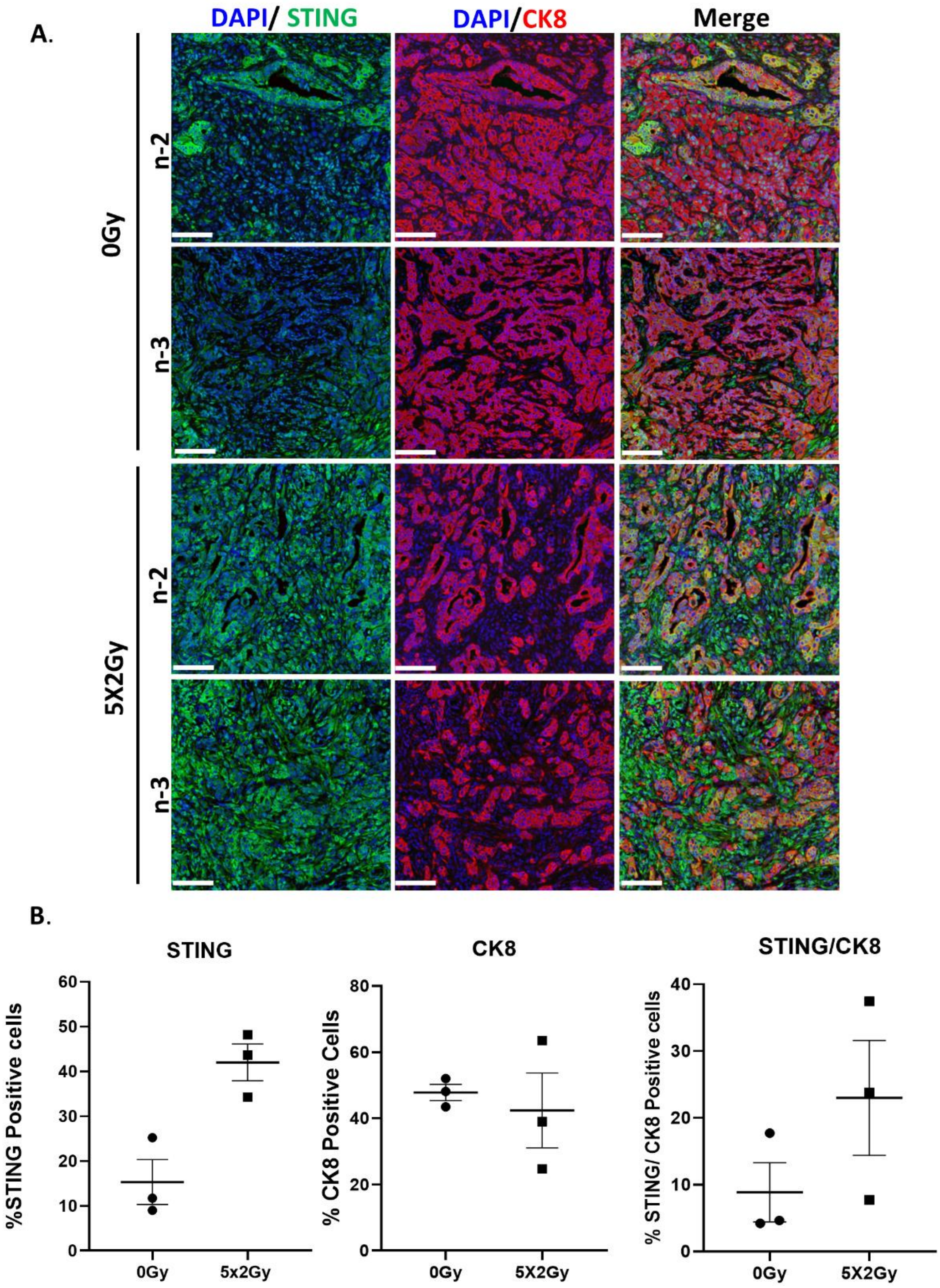

Figure S8. (**A**) Further images of STING immunohistochemical analysis (Green) revealed an upregulation of expression in response to radiotherapy (RT) primarily within glandular epithelium (defined as CK8+ cells), and as shown as co-localisation with CK8 (Red) in each tumour (3 mouse per group). STING was also upregulated in other cellular compartments within the TME (CK8-) areas suggestive of a global upregulation. DAPI nuclear counterstain shown in blue. Scale bar equals 100 µm. (**B**) Quantification of STING and CK8 staining showing an overall increase in the proportion of STING expressing positive cells in response to RT. Quantification was done using HALO® Image analysis software. Data expressed as a percentage positive relative to DAPI and expressed as mean ± SEM of n = 3 mice per group.

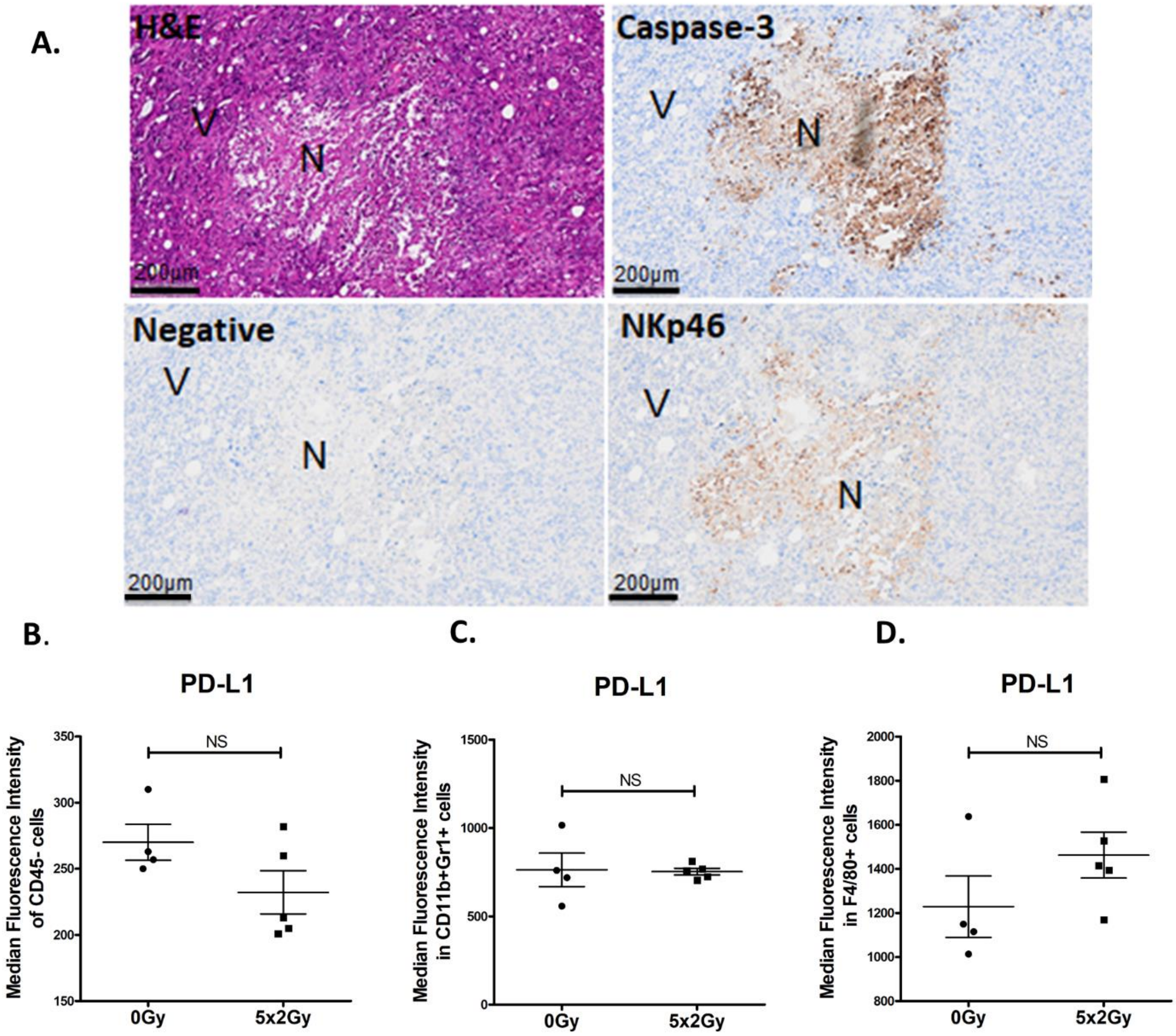

Figure S9. (**A**) Representative staining in corresponding sections of DVL3 tumour via H&E and immunohistochemistry for cleaved caspase-3 (Caspase-3) confirming necrosis in the irradiated tumour (top-panel). Immunohistochemical staining for NKp46 in the DVL3 tumours and corresponding no primary antibody negative control confirming NKp46 positive staining around the necrotic areas (bottom panel). Scale bar 200 µm. Expression of PD-L1 on DVL3 cells gated as CD45-ve cells (**B**), MDSCs which were gated as dual CD11b+, Gr1+ cells (**C**). Macrophages gated as CD45+/ F4/80+ (**D**) a week post-treatment with 5 fractions of 2Gy RT. Experimental groups contained 4–5 mice, data represents mean ± SEM. NS denotes non-significant, as determined by Mann Whitney statistical analysis.

Table S1. List of antibodies utilised for immunohistochemistry (IHC), Western Blotting, Immunocytochemistry (Cell IHC) and FACS sorting.

	Antigen	Clone	Species	Dilution	Manufacturer
Tissue IHC	CD8a	4SM15	Rat	1:100	eBioscience UK
	CD4	4SM95	Rat	1:100	eBioscience UK
	CD11b	EPR1344	Rabbit	1:8000	Abcam UK
	F4/80	D2S9R	Rabbit	1:100	New England Biolabs
	CK8	904804	Mouse	1:200	Biolegend
	CK5	ab52635	Rabbit	1:100	Abcam UK
	AR	M4070	Rabbit	1:200	Spring Bioscience
	KI67	ab15580	Rabbit	1:150	Abcam UK
	SMA	Ab124964	Rabbit	1:200	Abcam UK
	STING	D2P2F	Rabbit	1:100	New England Biolabs
	NKp46	AF2225	Goat	1:300	R&D
	Anti-Rabbit	BA-1000	Goat	1:500	Vector
	Anti- mouse	BA-9400	Rat	1:500	Vector
Western Blot	CK8	904804	Mouse	1:1000	BioLegend
	CK5	ab52635	Rabbit	1:10000	Abcam UK
	AR	ab9474	Mouse	1:2500	Abcam UK
	PTEN	9559S	Rabbit	1:1000	Cell signalling
	B-actin	ab6276	Mouse	1:2000	Abcam UK
	Anti-Rabbit	170-6515	Goat	1:3000	Bio-Rad
	Anti- mouse	170-6516	Goat	1:3000	Bio-Rad
Cell IHC	CK8	904804	Mouse	1:100	BioLegend
	CK5	ab52635	Rabbit	1:100	Abcam UK
	AR	ab74272	Rabbit	1:100	Abcam UK
	Anti-Rabbit 488	A11070	Goat	1:500	Life Technologies
	Anti- mouse 594	A11020	Goat	1:500	Life Technologies
FACS	EpCAM	130-102-969	Rat	1:100	Miltenyi Biotec
	CD4	RM4-5	Rat	1:100	Thermo Fisher, UK
	CD8	53-6.7	Rat	1:100	eBioscience UK
	F480	BM8	Rat	1:150	eBioscience UK
	CD11b	M1/70	Rat	1:150	BD Horizon UK
	Gr1 (Ly-6G)	RBC-8C5	Rat	1:100	eBioscience UK
	CD45	30-F11	Rat	1:100	eBioscience UK

Table S2. List of qRT-PCR primers.

Gene	Forward Primer	Reverse Primer
β-actin	AGTGTGACGTTGACATCCGT	CTTGCTGATCCACATCTGCT
CK8	CGGGGGATCCAACACTTTCA	GCTTCCCATCTCGGGTTTCA
CK5	CAGAGCTGAGGAACATGCAG	CACAAACTCATTCTCAGCCG
AR	GGGACCTTGGATGGAGAACT	GGTCTTCTGGGGTGGAAAGT

© 2020 by the authors. Submitted for possible open access publication under the terms and conditions of the Creative Commons Attribution (CC BY) license (http://creativecommons.org/licenses/by/4.0/).
